# Construction Notes

**Published:** 1904-01-02

**Authors:** 


### CONSTRUCTION NOTES.

Assouan's Hospital. — The Government has granted £10,000 for the erection of a new hospital at Assouan. There will be a special wing for infectious diseases.

Tantah.—The American Mission are completing a hospital at Tantah. Tantah is midway between Alexandria and Cairo, and so far the sick have been obliged to travel to either place in order to get into a hospital or nursing home.

Helouan Sanatorium.—The first sanatorium in the East has been erected at Helouan (near Cairo). The object of this establishment is for the admittance and treatment of tropical diseases, kidney cases, gout and rheumatism, as well as for tubercular cases not far advanced; the latter will occupy special rooms. Every appliance for the treatment of these cases has been provided — sand baths, electric and medicated baths. The reading-room and library, music-room and drawing-room, are in a block apart from the sleeping-rooms, while the dining-room occupies the space between the two blocks. The sanatorium is the property of a German company.

Improvements at the Royal Hants County Hospital.—Structural alterations have been proceeding at this hospital for some months past, and among other improvements is a new operating theatre on the top floor. In order to obtain a room of good proportions, only the lower part of the high mullioned window has been taken in; there is a skylight in the ceiling to admit additional light, and the upper room is used as an emergency store-room for surgical instruments. The floor is of parquet, and the walls white painted with a dado of green opaline tiles. Five coats of paint have been used, and the result is a peculiarly smooth and easily washable surface. The glass and iron furniture is by Allen and Hanbury, and the Doulton sinks have elbow taps. Heat is supplied by means of hot air coils, and there is space for an open fireplace. Close by, across a passage is the anæsthetising-room, formerly the nurses' sitting-room. Plain-surfaced swing doors are fitted to each room. Downstairs the bank has been put back several feet from the basement, so as to admit of a separate entrance to the out-patients' department, the central hall entrance having hitherto served the purpose. This department has been enlarged and surgeons' rooms added, comprising an ophthalmic room, single or double as required, sliding doors being provided, a dentist's room, and a Finsen lamp room, and there is also additional accommodation in the waiting hall. Electric light has been installed throughout the building.

